# Monitoring of the trough concentration of valproic acid in pediatric epilepsy patients: a machine learning-based ensemble model

**DOI:** 10.3389/fphar.2024.1521932

**Published:** 2024-12-18

**Authors:** Yue-Wen Chen, Xi-Kai Lin, Si Chen, Ya-Lan Zhang, Wei Wu, Chen Huang, Xin Rao, Zong-Xing Lu, Zhou-Jie Liu

**Affiliations:** ^1^ Department of Pharmacy, The First Affiliated Hospital, Fujian Medical University, Fuzhou, China; ^2^ Department of Pharmacy, National Regional Medical Center, Binhai Campus of the First Affiliated Hospital, Fujian Medical University, Fuzhou, China; ^3^ School of Mechanical Engineering and Automation, Fuzhou University, FuZhou, China; ^4^ Department of Pharmacy, The Second Affiliated Hospital, Fujian Medical University, Quanzhou, China

**Keywords:** machine learning, ensemble model, VPA trough concentration, pediatric epilepsy patients, SHAP

## Abstract

**Aims:**

Few personalized monitoring models for valproic acid (VPA) in pediatric epilepsy patients (PEPs) incorporate machine learning (ML) algorithms. This study aimed to develop an ensemble ML model for VPA monitoring to enhance clinical precision of VPA usage.

**Methods:**

A dataset comprising 366 VPA trough concentrations from 252 PEPs, along with 19 covariates and the target variable (VPA trough concentration), was refined by Spearman correlation and multicollinearity testing (366 × 11). The dataset was split into a training set (292) and testing set (74) at a ratio of 8:2. An ensemble model was formulated by Gradient Boosting Regression Trees (GBRT), Random Forest Regression (RFR), and Support Vector Regression (SVR), and assessed by SHapley Additive exPlanations (SHAP) analysis for covariate importance. The model was optimized for R^2^, relative accuracy, and absolute accuracy, and validated against two independent external datasets (32 in-hospital and 28 out-of-hospital dataset).

**Results:**

Using the R^2^ weight ratio of GBRT, RFR and SVR optimized at 5:2:3, the ensemble model demonstrated superior performance in terms of relative accuracy (87.8%), absolute accuracy (78.4%), and R^2^ (0.50), while also exhibiting a lower Mean Absolute Error (9.87) and Root Mean Squared Error (12.24), as validated by the external datasets. Platelet count (PLT) and VPA daily dose were identified as pivotal covariates.

**Conclusion:**

The proposed ensemble model effectively monitors VPA trough concentrations in PEPs. By integrating covariates across various ML algorithms, it delivers results closely aligned with clinical practice, offering substantial clinical value for the guided use of VPA.

## 1 Introduction

Epilepsy is a prevalent chronic neurological disorder, with a relatively high incidence rate among pediatric patients. The overall prevalence of epilepsy among children is approximately 151/100,000, a rate more than quadruple that of adults (Ding et al., 2021; Lin et al., 2021). At present, pharmacotherapy remains the cornerstone of seizure management (Liu et al., 2023). Among the available anti-seizure medications (ASMs), valproic acid (VPA) stands out as a broad-spectrum ASM, effective against various seizure types, including generalized, absence, partials, and myoclonic seizures. Its broad-spectrum efficacy has established VPA as a frontline clinical option for the treatment of pediatric epilepsy patients (PEPs) (Glauser et al., 2013). However, the therapeutic window for VPA, ranging from 50–100 mg/L, is relatively narrow and characterized by significant interindividual and intraindividual variability. These variability pose challenges for clinicians to manage dosages precisely and limiting the clinical application of VPA in treating PEPs (Johannessen Landmark et al., 2020).

In epilepsy management, medication safety is crucial. If VPA trough concentrations fall below the therapeutic range, seizures may not be adequately controlled, while exceeding this range may trigger severe adverse reactions in the hematopoietic and nervous systems, including anemia, thrombocytopenia, and ataxia, or even result in potentially lethal hepatotoxicity (Hernández García et al., 2023; Star et al., 2014). For adolescent girls, particularly those with childbearing potential, it is essential to reassess the risks associated with VPA exposure on an annual basis (Toledo et al., 2021). In the course of VPA dosage titration, it is imperative to align the trough concentration within the therapeutic range and to identify the most appropriate personalized concentration range for each PEP, for the therapeutic range is often more constrained, requiring further fine-tuning of blood concentrations to the desired therapeutic threshold within an ideal range (Johannessen and Landmark, 2008). Consequently, for PEPs necessitating prolonged therapeutic drug monitoring of VPA, the support of well-suited model-guided individualized VPA dosing tools can enhance the efficacy of monitoring VPA trough concentrations and adjusting VPA dosages, thereby enabling the establishment of more precise personalized treatment regimens.

Traditional methods for monitoring VPA trough concentrations, such as chromatography or immunoassay techniques, though accurate, are hampered by the problems of high cost and long detection cycles. In addition, these invasive detection methods can increase the psychological burden on PEPs, making them a less favorable option for those who require long-term monitoring. Therefore, model-guided individualized VPA dosing tools has currently surfaced as a heated research focus in the clinical arena, which may hold promises for reducing the number of invasive tests and producing an efficient and effective therapeutic range. Currently, the prevalent method for model-guided individualized dosing tools for VPA is the Nonlinear Mixed Effects Model (NONMEM). This method has been widely applied in population pharmacokinetics (popPK) studies (Gu et al., 2021). However, because it is based on parameter estimation, the operation and establishment process of the model is relatively complex, requiring a lot of time and efforts in constructing, validating, and optimizing the model. A recent systematic review meticulously curated and selected 10 published popPK models for PEPs, rigorously delineating their monitoring performance (Zhang et al., 2023). The findings indicated that the majority of these popPK models performed suboptimally in the context of pediatric epilepsy, which may potentially be attributed to the complexity of clinical data, insufficient sample sizes, deficiencies in model algorithms, and a lack of external data validation. Therefore, it is necessary to explore a more convenient and potent model for monitoring VPA trough concentrations in treating PEPs.

Currently, machine learning (ML) algorithms have exhibited significant potential in the medical field. They excel at processing complex and extensive clinical data and have been successfully applied in the monitoring of drug concentrations, including cyclosporine (Mao et al., 2022), mycophenolic acid (Shao et al., 2022), sertraline (Fu et al., 2024), and vancomycin (Huang et al., 2021). Moreover, preliminary studies have begun to emerge in the monitoring of VPA, such as the application of Fourier-transform infrared (FT-IR) spectroscopy combined with nonlinear support vector regression algorithms to construct a VPA monitoring model (El Orche et al., 2023) and the development of an XGBoost model to predict VPA trough concentrations by integrating covariates from various population pharmacokinetic models (Zhu et al., 2022a). Although ML-based VPA monitoring models have shown considerable potential in integrating real-world data and predicting VPA concentrations (Hsu et al., 2024), there are still limitations in external validation, comprehensiveness of evaluation metrics, data processing, and model interpretability. These are essential for guiding clinicians on which clinical indicators to monitor when treating PEPs, and they are what this study aims to address.

Ensemble models (Huang et al., 2021; Ma et al., 2022), by virtue of their ability to combine various ML algorithms to enhance the accuracy and robustness of monitoring models, are gaining increasing importance in the field of ML. Herein, we ranked the weights of each covariate across 3 ML models—Gradient Boosting Regression Trees (GBRT), Random Forest Regression (RFR), and Support Vector Regression (SVR) according to their SHapley Additive exPlanation (SHAP) (Ma et al., 2022). We then employed these algorithms to formulate the first ensemble model for monitoring VPA trough concentrations in PEPs. Our findings indicate that this ensemble model exhibits superior prediction performance, highlighting its significant potential in clinical applications.

## 2 Materials and methods

### 2.1 Patients and data

A retrospective study was conducted on PEPs treated with oral VPA from May 2016 to December 2023 at the First Affiliated Hospital of Fujian Medical University. The inclusion criteria were as follows: 1) epilepsy patients; 2) age ≤14 years old; 3) oral VPA treatment (solution or extended-release tablets); 4) VPA trough concentration samples collected within 30 min before the next dose after reaching a steady concentration. The exclusion criteria included: 1) patients with incomplete clinical medical records; 2) patients co-administered with drugs such as carbamazepine, phenobarbital, or carbapenems; 3) patients with other systemic severe diseases. The clinical data of PEPs were obtained from the hospital’s electronic medical record information system (EIS), which included VPA trough concentrations, VPA dosage form and daily doses, concomitant medications (including levetiracetam, oxcarbazepine, lamotrigine, perampanel, clonazepam, or topiramate), demographic information (gender, age, weight), and laboratory parameters [alanine aminotransferase (ALT), aspartate aminotransferase (AST), alkaline phosphatase (ALP), urea (UREA), uric acid (UA), creatinine (CREA), cystatin C (Cys-C), albumin (ALB), globulin (GLO), white blood cell count (WBC), neutrophil count (NEUT), platelet count (PLT), and red blood cell count (RBC)].

### 2.2 Measurement of VPA trough concentration

The VPA trough concentration was measured with an automated biochemical analyzer Viva-ProE (Siemens Medical Diagnostic Products Ltd.). After a steady VPA concentration was secured, blood plasma samples were collected 30 min before the next dose. Subsequently, the plasma samples were pre-processed and the supernatant was collected. Finally, the VPA trough concentration was determined by the Enzyme Multiplied Immunoassay Technique (EMIT). The detection limit ranged from 10.0 to 150.0 mg/L. As part of the validation sets, the VPA trough concentrations in the external hospital dataset also met the requirements of this detection standard.

### 2.3 Data collection and processing

Data cleansing included removing missing values (delete samples containing missing values directly), correcting outliers, assigning values to qualitative variables, and standardizing formats, we acquired a comprehensive dataset of 366 samples by 20 variables (366 × 20). Subsequently, Spearman correlation analysis and multicollinearity testing were rigorously performed to select the target variable and relevant key covariates, ultimately producing a complete dataset containing 366 × 11. In this refined dataset, VPA trough concentration was designated as the target variable, with covariates standardized for different dimensions by min-max normalization. To ensure the fairness of the model comparison, we labeled the sample numbers for this dataset (366 × 11) and then randomly divided it into a training set and a testing set in an 8:2 ratio, and performed cross-validation on the dataset to assess and ensure that the trained models posses a certain level of generalizability. Subsequently, we saved the randomly divided sample numbers to ensure that all models were trained and tested on the same datasets, thereby making the performance comparison more accurate and reliable. Moreover, to bolster the model’s reliability, we prospectively collected two independent external datasets as our validation set, including an in-hospital dataset (n = 32) and an external hospital dataset (n = 28). Both datasets contained complete clinical parameters of PEPs. In this study, each measurement of VPA trough concentration was regarded as an independent data point for independent analysis.

### 2.4 Modeling and validation

#### 2.4.1 Algorithm selection

We conducted an in-depth assessment of the linear correlation between VPA trough concentrations and relevant covariates (Supplementary Table S1). The analysis revealed insignificant linear relationships among these variables, with low correlation coefficients. In light of these findings, we decided to employ three advanced nonlinear ML algorithms (GBRT, RFR, and SVR) to construct more accurate monitoring models. These nonlinear algorithms can capture complex patterns and nonlinear relationships within the data, thereby providing more robust modeling capabilities for the monitoring of VPA trough concentrations [sklearn module package in the Python programming language (version 3.10.9)].

#### 2.4.2 Model evaluation metrics

Regression model evaluation methods in ML are primarily used to measure the degree of fit of the model to the dataset and its monitoring power. We used metrics such as R-squared (R^2^), Mean Absolute Error (MAE), and Root Mean Squared Error (RMSE) for evaluation. R^2^ measures the degree to which the model explains the variability in the data, with a range from 0 to 1, where the closer the value is to 1, the stronger the explanatory power of the model and the better the fit. y^0^ represents the observed values and y^p^ the predicted values. The fit of the model improves as the values of MAE and RMSE decrease. Additionally, relative accuracy indicates that the predicted concentration is within ±30% of the observed concentration, while absolute accuracy indicates that the predicted concentration is within ±15 mg/L of the observed concentration (Hsu et al., 2024; Tang et al., 2023). The formulas for the above metrics were as follows:

$$R^{2}\left(y^{0},y^{p}\right)=1-\frac{\sum_{i=1}^{N}{\left(y_{i}^{0}-y_{i}^{p}\right)}^{2}}{\sum_{i=1}^{N}\left(y_{i}^{0}-\bar{y^{0}}\right)}$$

$$\bar{y^{0}}=\frac{1}{N}\sum_{i=1}^{N}y_{i}^{0}$$

$$MAE\left(y^{0},y^{p}\right)=\frac{1}{N}\sum_{i=1}^{N}\left|y_{i}^{0}-y_{i}^{p}\right|$$

$$RMSE\left(y^{0},y^{p}\right)=\sqrt{\frac{1}{N}\sum_{i=1}^{N}{\left(y_{i}^{0}-y_{i}^{p}\right)}^{2\;}}$$

#### 2.4.3 Formulation and validation of the ensemble model

Random search is a strategy used to optimize the hyperparameters of ML-based models. It involves combinations of randomly-selected hyperparameters within a specified range for model training and evaluation to find the optimal or near-optimal parameter settings (Pérez-Padilla et al., 2024). In this study, we used the random search module in Python to draw 100 hyperparameter sample points within the selected range as a set of candidates and performed 5-fold cross-validation. After multiple calculations, the near-optimal hyperparameters were determined for the three algorithms in this experiment: GBRT: n_estimators (68), min_samples_split (13), min_samples_leaf (24), max_depth (4), learning_rate (0.1); RFR: n_estimators (152), min_samples_split (15), min_samples_leaf (9), max_depth (7); SVR: kernel (rbf), C (19.1), gamma (6.8).

**FIGURE 1:**
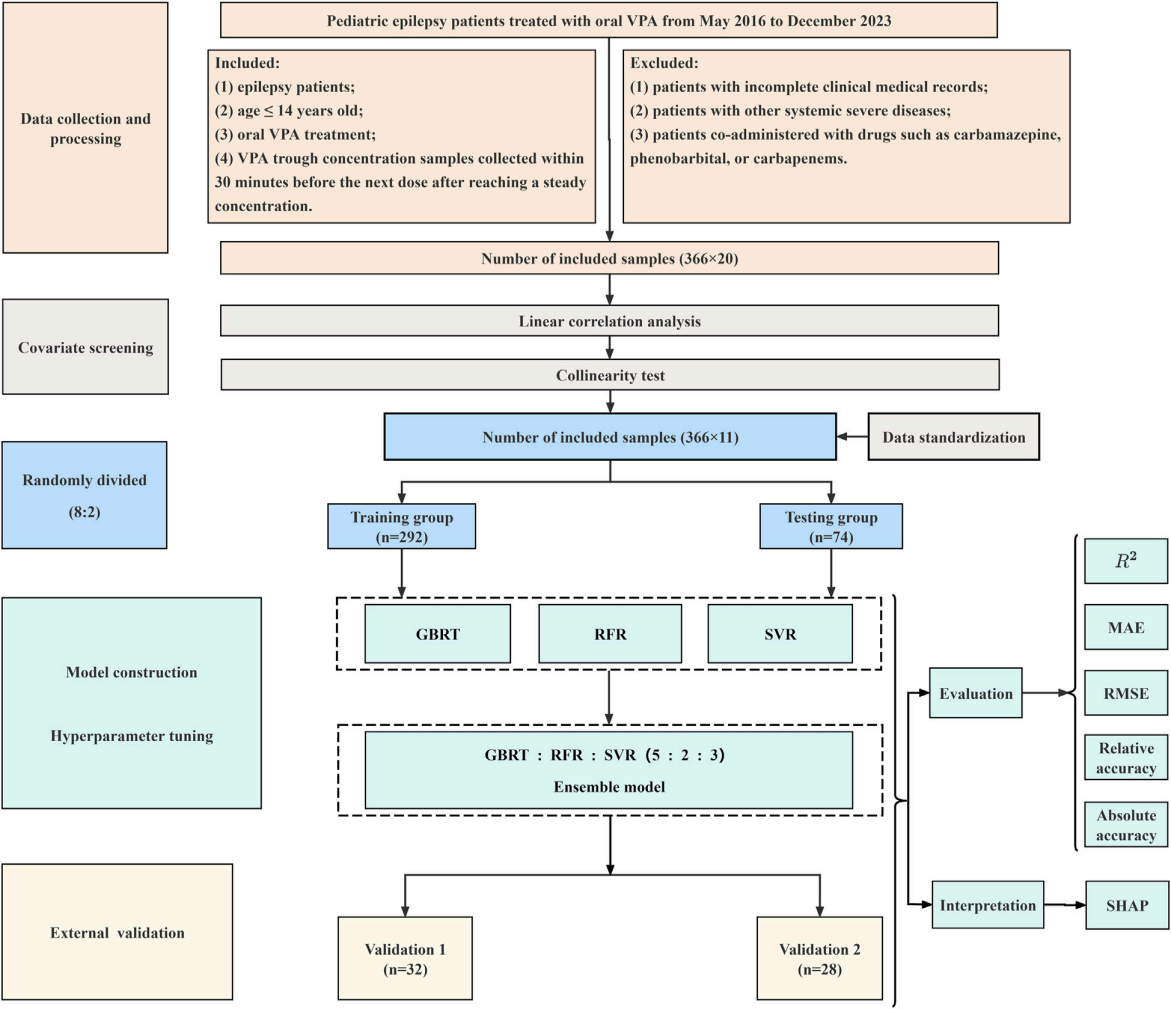
Flowchart of the ensemble model for monitoring VPA trough concentrations in PEPs by the 3 ML algorithms.

These three optimal ML-based models were then selected and their weight ratios of the R^2^ values were adjusted. Based on the automatic computation of ML, the optimal ensemble model was ultimately determined. Finally, the monitoring performance of the ensemble model was prospectively confirmed with the two independent external datasets. The flowchart for formulaing the ensemble model of VPA trough concentrations in treating PEPs by the 3 ML algorithms is shown in Figure 1.

### 2.5 Model interpretation

SHapley Additive exPlanations (SHAP) is based on the concept of the Shapley value from game theory. The Shapley value is a solution in cooperative game theory for fairly allocating the total payoff from cooperation, quantifying the contribution of each player (a feature of ML) to the total payoff (the model’s monitoring outcome). SHAP offers a variety of visualization tools for ML models, such as SHAP value plots, dependence plots, decision plots, and summary plots, which facilitates an intuitive understanding of the model’s monitoring process and the impact of features (Li et al., 2024; Zhu et al., 2022b). In this study, we implemented SHAP using the Python package (version 0.41.0).

### 2.6 Statistical analysis

Data processing was performed using IBM SPSS software (version 25.0). In terms of statistical description, for categorical variables, we expressed the data as counts n (%) and analyzed distribution differences by the chi-square test. For continuous variables, we presented the data as the mean ± standard deviation (SD) and, based on the characteristics of the data distribution, assessed non-normally distributed data by the Mann-Whitney U test and normally distributed data by the independent samples *t*-test. When exploring the correlation between variables, we adopted the Spearman correlation coefficient. A value of *p* < 0.05 was deemed statistically significant.

### 2.7 Ethic statement concerning actual patients

This study strictly adhered to the ethical guidelines set forth in the Declaration of Helsinki, ensuring the morality and legality of the research. Our research plan was officially approved by the Ethics Committee of the First Affiliated Hospital of Fujian Medical University [Ethics No.: (2022)325]. Informed consent has been waived in the ethical approval document.

## 3 Results

### 3.1 Baseline information of PEPs

In this retrospective analysis, we utilized 366 VPA trough concentrations derived from 252 PEPs, with VPA trough concentration as the target variable. After the dataset was cleaned, a dataset of 366 rows by 20 columns was obtained. Subsequently, the pertinent covariates were meticulously selected by Spearman correlation analysis and multicollinearity testing. The Spearman correlation analysis revealed age, ALT, UREA, CREA, RBC, PLT, VPA dosage form, VPA daily dose, and Weight as significant covariates (*p* < 0.05 for all) (Supplementary Table S1). ALB, a crucial hematological marker affecting VPA drug concentrations, was also included in the model despite its lack of strong correlation in this data analysis. Further statistical analysis reported a pronounced multicollinearity between age and weight (Supplementary Table S2). Despite the presence of multicollinearity between age and weight, these variables were retained in the model due to their significance in clinical decision-making for PEPs. Consequently, the final dataset comprised 366 samples by 11 variables (366 × 11). This dataset was then randomly partitioned into a training set (292 cases) and a testing set (74 cases) at an 8:2 ratio. There were no statistical differences in the baseline information of the PEPs and the variables between the training and test groups (*p* > 0.05 for all variables, with the exception of ALB) (Table 1).

**TABLE 1:** The description of the baseline PEP information.

Variables	Values		*p*-value
	Training group (n = 292)	Testing group (n = 74)	
VPA trough concentration (mg/L)	63.5 ± 22.7	64.0 ± 17.3	0.887^a^
Age (months)	92.8 ± 41.7	92.3 ± 40.6	0.424^a^
ALT (IU/L)	13.1 ± 10.5	10.9 ± 5.1	0.117^a^
UREA (mmol/L)	4.3 ± 1.0	4.2 ± 1.0	0.818^a^
CREA (μmol/L)	37.1 ± 10.3	39.0 ± 9.2	0.051^a^
RBC (10^12^/L)	4.6 ± 0.4	4.6 ± 0.4	0.600^a^
PLT (10^9^/L)	256.6 ± 67.9	262.2 ± 60.4	0.404^a^
VPA daily dose (mg)	535.4 ± 230.1	501.9 ± 198.6	0.395^a^
Weight (kg)	28.0 ± 11.4	29.0 ± 11.8	0.518^a^
ALB (g/L)	44.8 ± 2.6	45.6 ± 2.4	0.021^b^
Dosage form (n, %)			0.714^c^
Sustained-release tablet	152 (52.1%)	37 (50%)	
Oral solution	140 (47.9%)	37 (50%)	

VPA, valproic acid; ALT, alanine aminotransferase; UREA, urea; CREA, creatinine; RBC, red blood cell count; PLT, platelet count; ALB, albumin.

^a^ Mann-Whitney U test.

^b^ Independent samples *t*-test.

^c^ Chi-squared test.

### 3.2 The establishment of the three ML-based covariate models

We initially constructed independent covariate models for monitoring VPA trough concentrations in PEPs by the 3 ML algorithms (GBRT, RFR, and SVR). According to the results from both the training and testing groups (Table 2), all three models exhibited satisfactory monitoring performance, with the GBRT model showing the best results, featuring higher accuracy rates and R^2^ and a lower RMSE (Supplementary Figures S1–S3).

**TABLE 2:** Comparative performance of the three ML-based covariate models in the training and testing group.

Group	Model	MAE	RMSE	*R* ^*2*^	Relative accuracy^a^	Absolute accuracy^b^
Training	GBRT	11.08	14.43	0.60	82.2%	75.3%
	RFR	12.88	16.87	0.45	77.4%	67.1%
	SVR	10.06	16.32	0.48	82.2%	75.0%
Testing	GBRT	10.06	12.57	0.47	85.1%	82.4%
	RFR	10.50	12.78	0.45	85.1%	75.7%
	SVR	11.26	13.75	0.36	81.1%	73.0%

GBRT, gradient boosted regression trees; RFR, random forest regression; SVR, support vector regression.

^a^ Relative accuracy indicates that the predicted trough concentration was within ±30% of the observed trough concentration.

^b^ Absolute accuracy indicates that the predicted trough concentration was within ±15 mg/L of the observed trough concentration.

### 3.3 Interpretation of the ML-based covariate models

**FIGURE 2:**
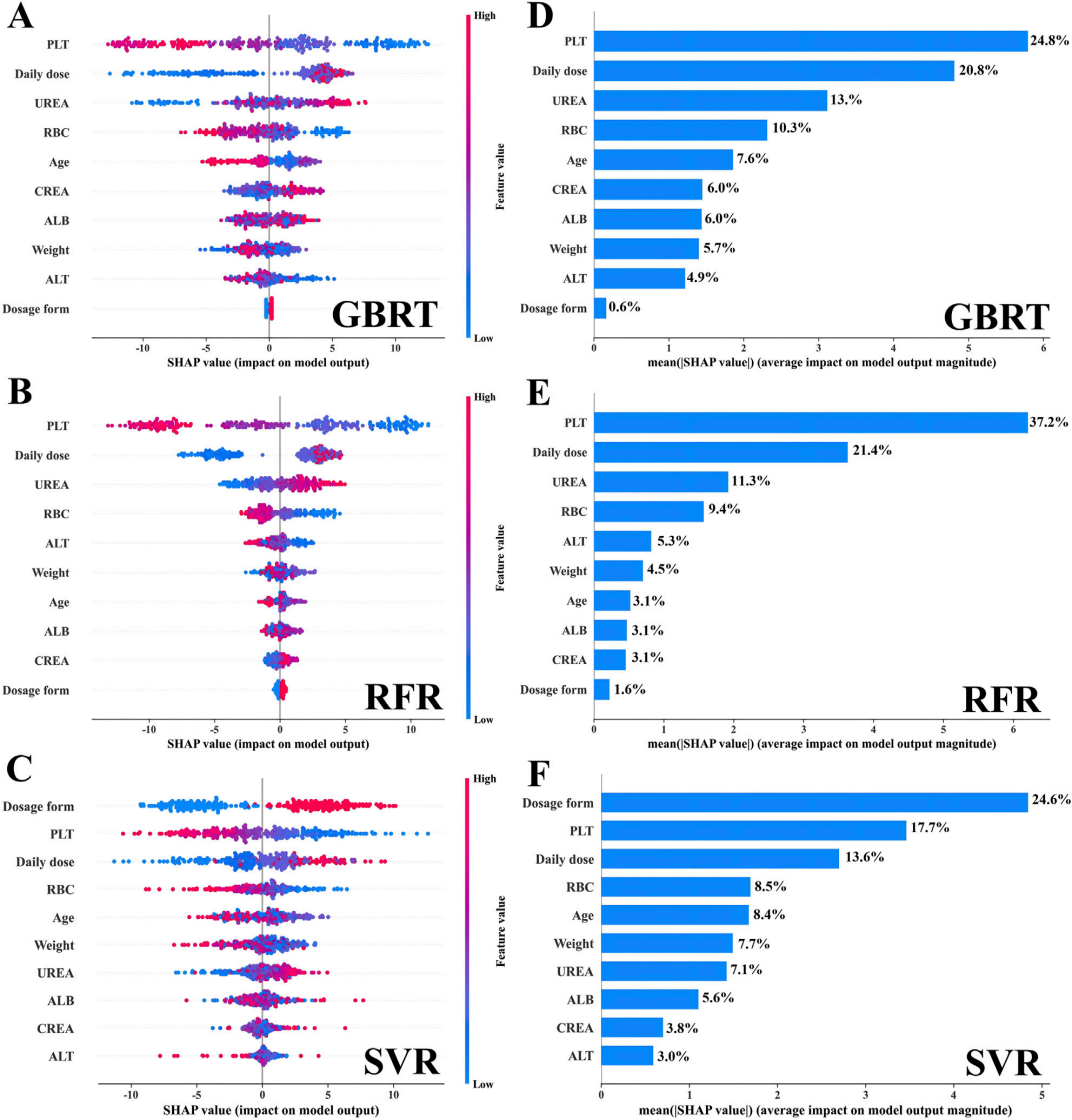
The interpretation of the three models by SHAP. **(A–C)** The SHAP summary plot for the ten covariates across the three models. The SHAP values (x-axis) serve as a unified measure of the impact of the response variable in the model. In the variable weight ranking, the attributes for all patients contributing to the outcome are plotted with dots of different colors, where red (blue) dots represent high (low) values, respectively. **(D–F)** The weight ranking of the ten variables according to the mean (|SHAP value|).

In this study, we conducted a visual analysis of the influencing factors in the three ML-based covariate models by SHAP. Based on the selected ten covariates, the SHAP plot displayed the contribution of each covariate to the model monitoring, ranked from high to low. As shown in Figures 2A–C, the color of the dots represents the feature values of each variable, with larger feature values being redder and smaller feature values bluer. Each feature value of a variable corresponded to a SHAP value (on the x-axis), with negative influences indicated on the left side of the origin, positive influences on the right side, and little or no influence in the center. The average of the absolute SHAP values for each relevant covariable was calculated. For the GBRT and RFR models (Figures 2D, E), the top four covariates were PLT, daily dose, UREA, age, and RBC, indicating their importance in monitoring VPA trough concentrations, with dosage form ranked 10th (|SHAP value| < 0.5) and having the least impact on monitoring VPA trough concentrations; for the SVR model (Figure 2F), the top four covariates were dosage form, PLT, daily dose, and RBC, while UREA was ranked 7th (|SHAP value| < 1.5).

**FIGURE 3:**
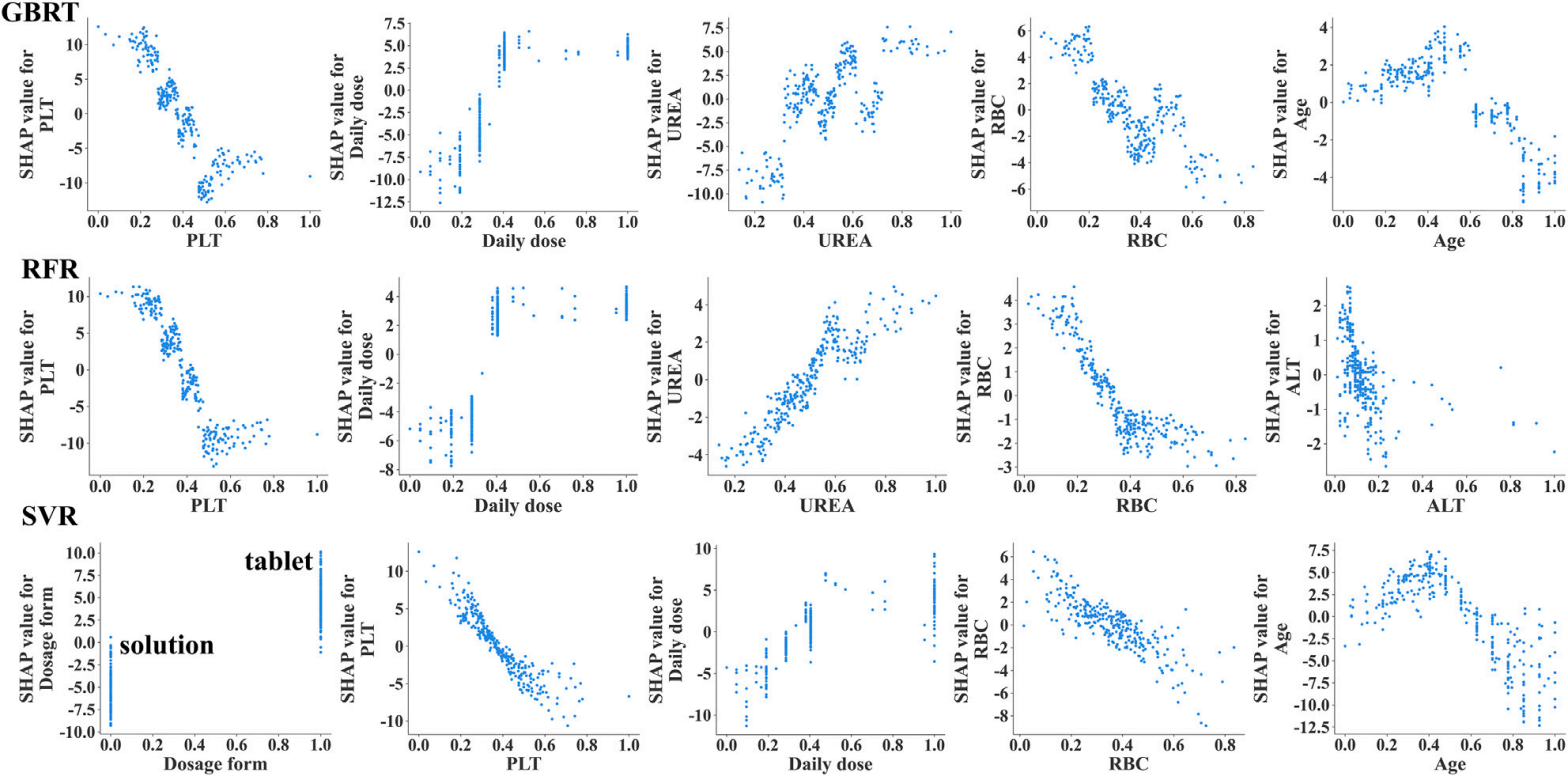
The SHAP dependency plots illustrating the importance of the top 5 variables in the 3 ML models (GBRT, RFR, SVR). The SHAP dependency plots demonstrate how the relevant variables influence the output of the monitoring model. When the SHAP value for a specific covariate exceeds 0, it indicates an increase in VPA trough concentration. VPA dosage form, 0 indicates oral solution, 1 indicates sustained-release tablet.

**FIGURE 4:**
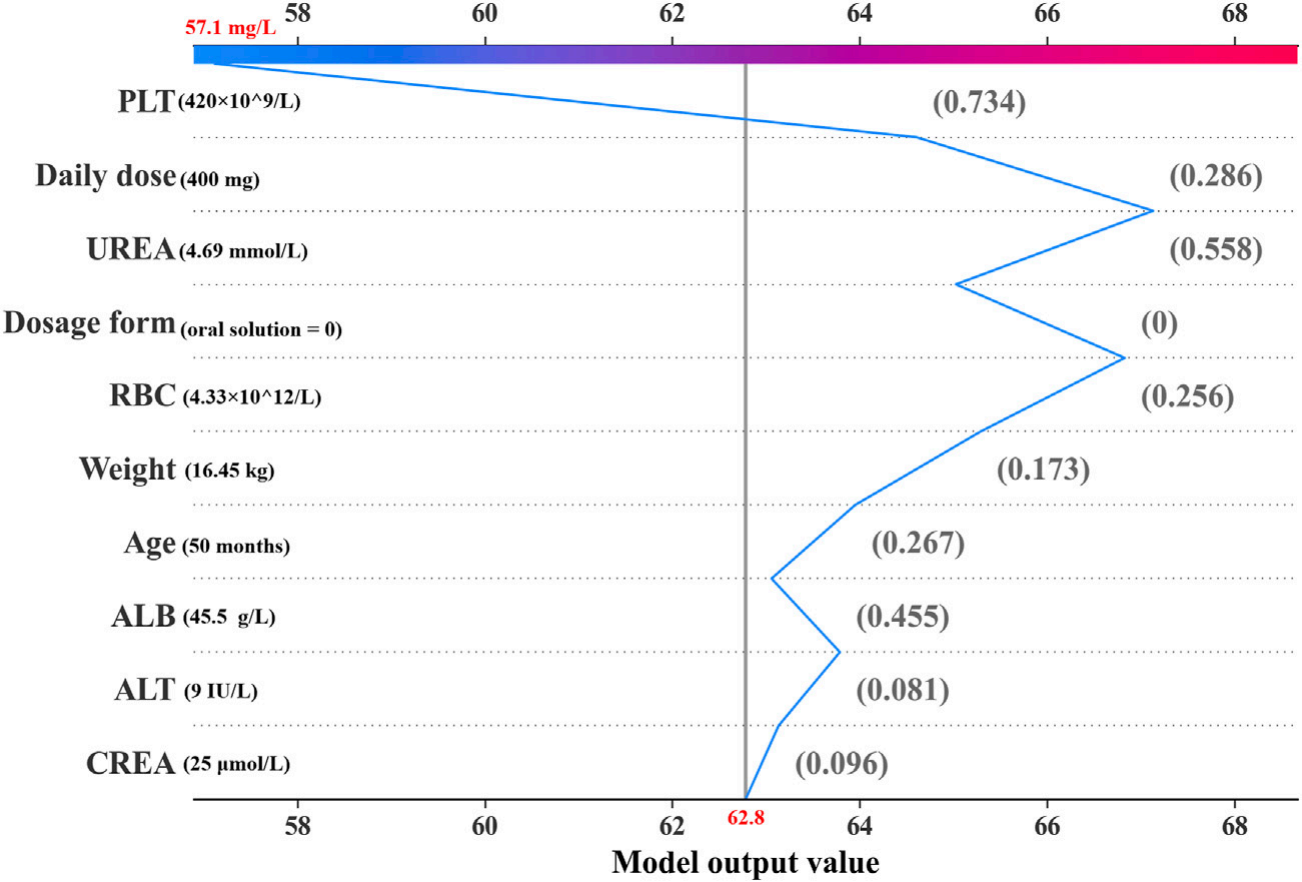
Decision plot for an individual sample within the ensemble model. The gray vertical line in the middle of the decision graph marks the base value of the ensembl model (62.8 mg/L). The colored broken line is the prediction line representing the prediction process. This prediction line indicates the numerical contributions of the covariates on the predicted result, which are defined as SHAP values. The normalized values of covariates are placed next to the prediction line for reference. Starting from the bottom and ending at the top of the graph, the prediction line shows how the prediction result (57.1 mg/L) is calculated from the base value and the accumulating SHAP values.

Additionally, we depicted the SHAP dependence plots for the top 5 variables of the three algorithms (Figure 3). These results indicated that in the GBRT and RFR models, a higher VPA daily dose and a higher level of UREA, along with lower PLT and RBC, were associated with higher VPA trough concentrations; in the SVR model, a higher VPA daily dose and sustained-release tablet (dosage form), along with lower PLT and RBC, were associated with higher VPA trough concentrations. To better understand the decision-making process of the ensemble model, we plotted the decision-making plot for individual sample within the ensemble model (Figure 4). Of note, UREA and VPA dosage form exhibited a consistent significant variability across models (Figure 3), prompting us to formulate an ensemble model designed to maximize the impact of each covariate and enhance the accuracy of monitoring.

### 3.4 Formulation and validation of the ensemble model

**FIGURE 5:**
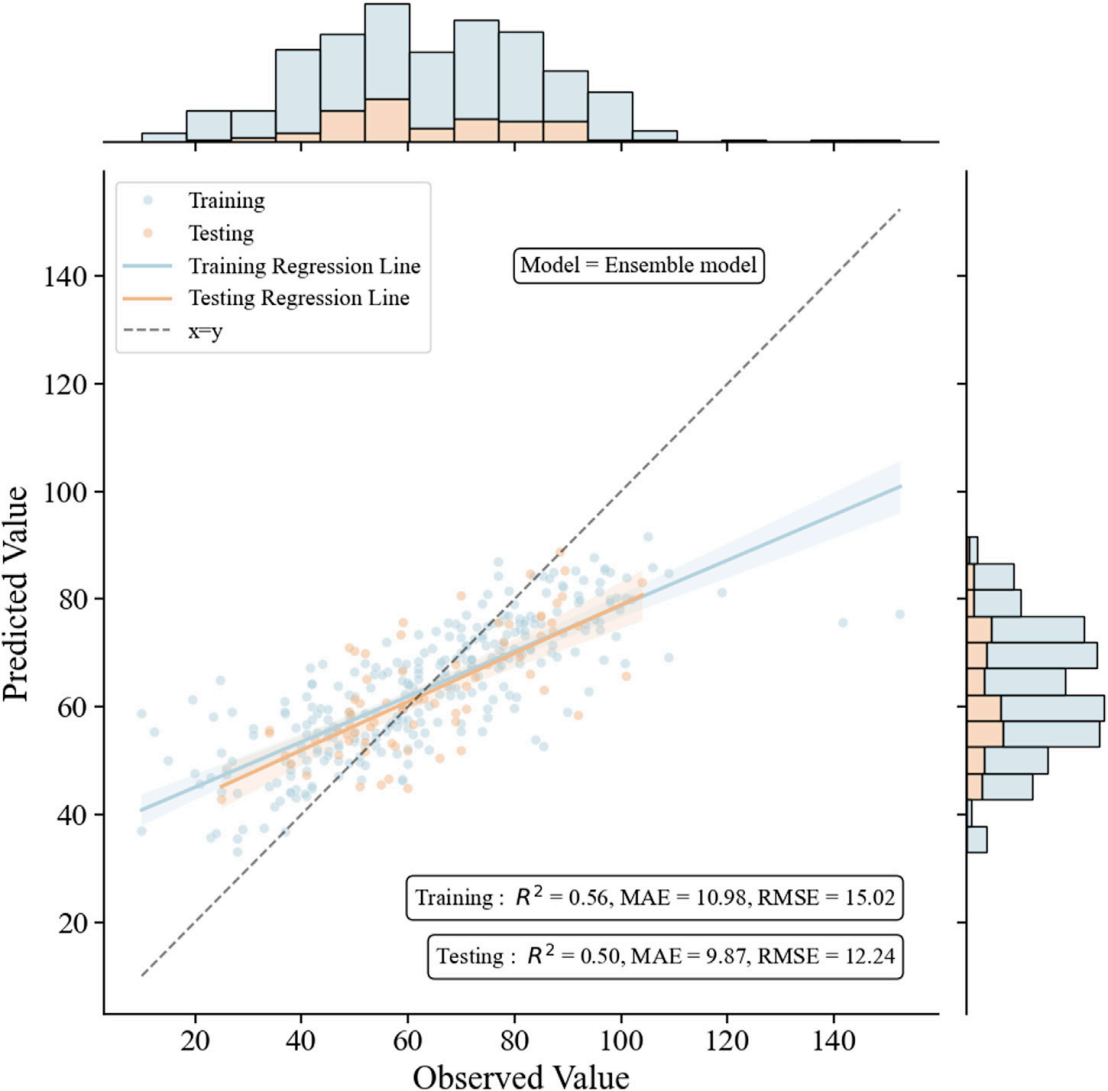
The performance of the ensemble model in the training and the testing groups. The figure includes scatter plots, regression lines, and histograms to intuitively compare the relationship between observed and predicted values, and to judge the fitting effect of the model through the R^2^ value and the diagonal line.

**FIGURE 6:**
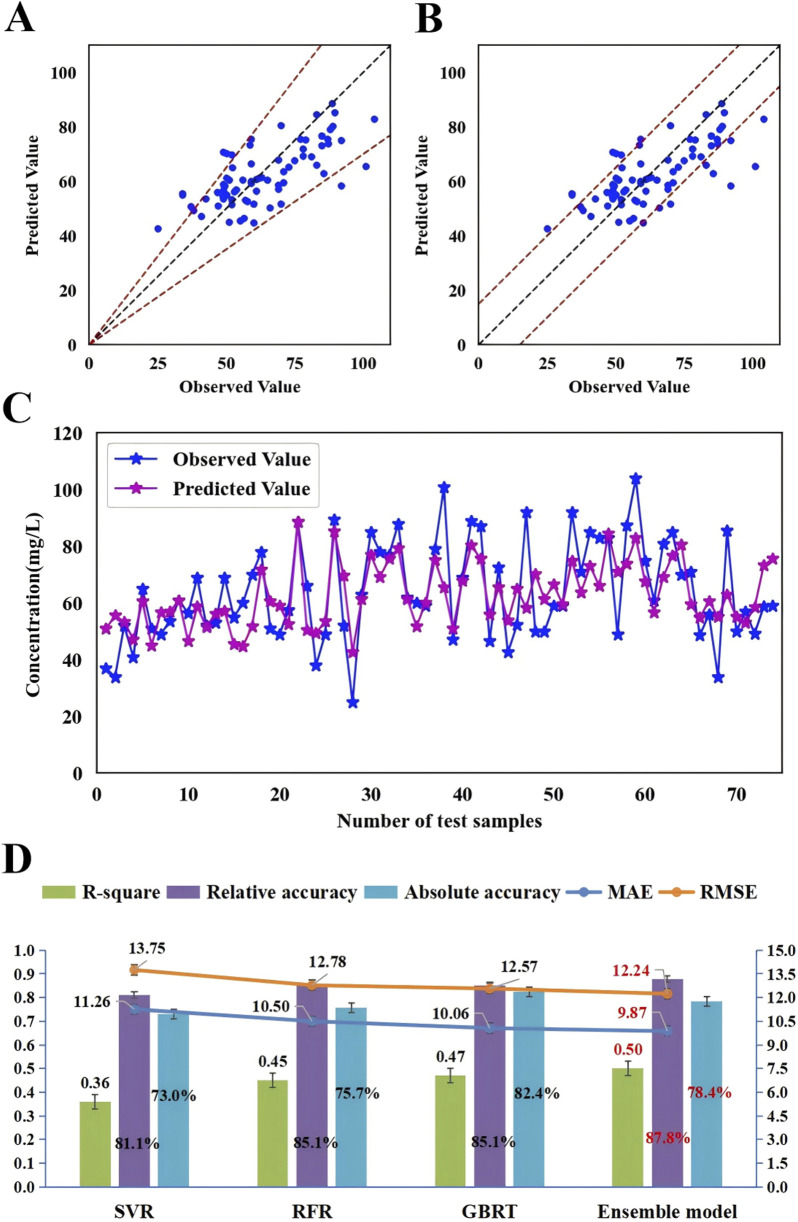
The accuracy and prediction plots of the ensemble model and a comparison of the monitoring performance of the ensemble model with that of three independent covariate models. **(A)** The blue dots represent the testing sample, with observed values on the x-axis and predicted values on the y-axis. The blue dots between the dotted lines indicate that the predicted values were within ±30% of the observed values (relative accuracy) and **(B)** the blue dots between the dotted lines indicate that the predicted values were within ±15 mg/L of the observed values (absolute accuracy). **(C)** The blue dots indicate the observed values and purple dots indicate the predicted values. The samples on the x-axis are ordered according to age, increasing from left to right in ascending order. **(D)** Comparison of monitoring performance of the ensemble model with that of SVR, RFR and GBRT.

To further enhance the monitoring performance of the model, we employed 3 ML algorithms to formulate the first ensemble model for monitoring VPA trough concentrations in PEPs. This model was designed to achieve the highest R^2^ value, relative accuracy, and absolute accuracy. Based on the R^2^ performance of these three algorithms (GBRT, RFR, and SVR) on the training set, we assigned an initial random uniform distribution range for the weight of each model. Specifically, the model with the highest R^2^ value was given a weight range of w1 (0.4–0.6), followed by w2 (0.2–0.4), and the model with the lowest R^2^ value was assigned a weight of w3 = 1–w1–w2. The final prediction of the ensemble model is obtained by multiplying the predictions of these three algorithms by their respective weights and summing them up. After several iterations of calculations, the optimal weights for these three algorithms (GBRT, RFR, and SVR) were ultimately determined to be 0.5, 0.2, and 0.3, respectively. As depicted in Figure 5, this ensemble model exhibited consistent performance in both the training and testing sets, indicating an excellent model fit. Moreover, when compared with the three independent covariate models, this ensemble model demonstrated superior monitoring performance, reporting the highest R^2^ (0.50), relative accuracy (87.8%), and absolute accuracy (78.4%), and the lowest MAE (9.87) and RMSE (12.24) (Figure 6; Supplementary Figures S1–S3).

**FIGURE 7:**
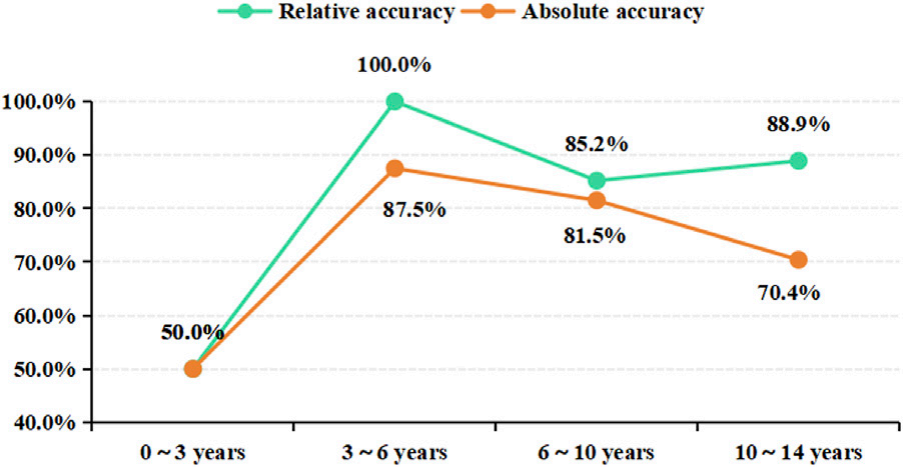
The monitoring results of the ensemble model for different age subgroups. Age subgroup on the x-axis, prediction accuracy on the y-axis.

In an in-depth analysis of the ensemble model, particular attention was paid to the monitoring performance across various age subgroups. The analysis revealed that among the PEPs aged less than 3 years, the performance of the model was suboptimal, with a relative and absolute accuracy of only 50.0% (Figure 7). Given the insufficient sample size of PEPs under 3 years old and over 10 years old in this study, we also performed stratified modeling for PEPs aged between three and 10 years. The results (Supplementary Tables S3, S4) indicate that both the three ML-based covariate models and the ensemble model demonstrate favorable predictive performance, as evidenced by further improvements in R^2^ values and reductions in MAE and RMSE. To further ascertain the stability and generality of the ensemble model, two independent external datasets were prospectively collected as validation cohorts. The results showed that the monitoring performance of the test group and that of the validation group were highly consistent, thereby fully validating the reliability and effectiveness of the ensemble model in practical applications (Table 3; Supplementary Table S5).

**TABLE 3:** The model performance metrics of the ensemble model.

Group	MAE	RMSE	*R* ^*2*^	Relative accuracy	Absolute accuracy
Training	10.98	15.02	0.56	82.2%	74.7%
Testing	9.87	12.24	0.50	87.8%	78.4%
Validation 1	9.89	13.26	0.41	81.3%	78.1%
Validation 2	9.49	12.31	0.46	82.1%	71.4%

Validation 1, 32 in-hospital data samples.

Validation 2, 28 out-of-hospital data samples.

## 4 Discussion

The ensemble model is a robust ML technique (Supplementary Table S6) that enhances overall monitoring performance by combining the predictive characteristics of multiple models. This approach is recognized for reducing overfitting and enhancing generality, and often outperforms a single model in various scenarios (Ma et al., 2022). In this study, we developed the first ML-based ensemble model for monitoring VPA trough concentration in PEPs, with the goal of maximizing the R^2^ value, relative accuracy, and absolute accuracy to optimize the performance. We selected three outstanding algorithms (GBRT, RFR, and SVR) and reallocated the weights of their R^2^ values to reflect their importance and contribution in the ensemble model. By utilizing advanced ML techniques, we achieved automatic computation and model optimization, ultimately developing an ensemble model with an optimal comprehensive performance, with the R^2^ weight ratio of GBRT, RFR, and SVR optimized at 5:2:3. Compared with an individual covariate model, this ensemble model demonstrated superior advantages across multiple dimensions and was further fully validated by two independent external sample groups (in-hospital and out-of-hospital datasets), demonstrating good generality.

SHAP plot visualization analysis plays a crucial role in the construction of ML models. It not only assigns a specific set of SHAP values to each covariate, clearly showing their importance and direction of influence on the monitoring results of the model, but also potentially explains the correlation between each covariate and its clinical significance (Cheng et al., 2023). Existing studies have shown that the daily dose and dosage form of VPA significantly affect VPA blood concentration (Zhu et al., 2022a; Xu et al., 2020). Consistently, in our study, the VPA daily dose ranked high in the SHAP visualization analysis [GBRT (second, 20.8%), RFR (second, 21.4%), and SVR (third, 13.6%)] (Figure 2); the VPA dosage form ranked first (24.6%) in SVR; and both the VPA daily dose and dosage form were positively correlated with VPA trough concentration (Figure 3), indicating that they are among the most important covariates. Therefore, in the clinical medication process, especially during a switch between VPA dosage forms (oral solution and sustained-release tablet), due to the differences in drug release and absorption between the two dosage forms, regular monitoring of VPA blood concentration in PEPs is necessary in case of VPA dosage adjustment.

VPA is commonly associated with hematological adverse reactions, particularly with thrombocytopenia and red blood cell dysplasia, which may be related to its impact on bone marrow hematopoietic function (Langlie et al., 2022; Acharya and Bussel, 2000). It is important to note that high concentrations of VPA may not only reduce platelets but also inhibit platelet aggregation, fibrinogen, and other factors, which can increase the bleeding risk in patients with epilepsy (Kumar et al., 2019; Beydoun et al., 1997). Additionally, although there is no evidence that VPA directly acts on RBCs, available data confirm that VPA may interact with RBCs through different mechanisms, including affecting the distribution of drugs within RBCs and enhancing the expression of erythropoietin (EPO) protein, which may affect RBC production (Mancl and Gidal, 2009; Rubiyana et al., 2020). This study showed that VPA trough concentration was inversely proportional to PLT and RBC levels (Figure 3), meaning that high VPA concentrations are more likely to cause a decrease in PLT and RBC, inducing the occurrence of thrombocytopenia and anemia, which is consistent with the existing research (Nasreddine and Beydoun, 2008; Nasreddine et al., 2022; Ma et al., 2019). Furthermore, SHAP plot analysis ranked RBC relatively high (consistently ranked fourth across the three models, with a contribution of 8.5%–10.3%), and PLT as one of the important covariates in different individual covariate models (Figure 2): GBRT (first, 24.8%), RFR (first, 37.2%), and SVR (second, 17.7%). For ALB, another important hematological indicator affecting VPA drug concentration, patients with hypoalbuminemia (below 35 g/L) may have significantly-increased free VPA concentrations (Drisaldi et al., 2019). However, the PEPs data analysis in this study showed that ALB fell within the normal range (44.98 ± 2.58 g/L) and was not correlated with VPA trough concentration (*r* = −0.026, *p* > 0.05). This phenomenon can explain why the weight of ALB in monitoring VPA trough concentration was relatively low. The above results indicate that the monitoring model for VPA trough concentration constructed in this study has strong clinical relevance. Therefore, in clinical practice, for PEPs receiving VPA, regular blood tests should be recommended to monitor potential adverse reactions in the hematological system, especially for PEPs with trough concentrations above the therapeutic window, in order to adjust VPA dosage in a timely manner.

CREA and UREA are two key clinical indicators for assessing kidney function. In the SHAP plot analysis, CREA ranked low in the three independent covariate models, indicating its relatively small impact on the model output. In contrast, UREA ranked third in the GBRT and RFR algorithms, highlighting the key role of UREA in VPA trough concentration (Figure 2). Existing studies have confirmed that VPA concentration is positively correlated with the severity of renal tubular damage (Knights and Finlay, 2014; Mazaheri et al., 2011) and that CREA and UREA levels are also positively correlated with VPA concentration (Yang et al., 2020). These findings suggest that increased levels of CREA and UREA generally indicate impaired kidney filtration function and can reflect VPA blood concentration levels to some extent. Changes in UREA levels need to be comprehensively assessed in conjunction with other indicators and clinical conditions. In some cases, UREA may not be as sensitive as CREA, leading to the clinical practice of using both to assess kidney function. For PEPs who use VPA on a long-term basis or have specific risk factors, it is essential to prescribe a regular monitoring of the kidney function and adjust the treatment plan correspondingly (Mazaheri et al., 2011). Therefore, incorporating CREA and UREA as covariates in our model has significant clinical implications for the effective management of VPA in treating PEPs.

Studies have shown that genetic variations in *CYP2C9*, *CYP2C19*, *CYP2A6*, and *CYP2B6* affect the *in vivo* concentrations of VPA and its metabolites (Zhao et al., 2020). The risk of hepatotoxicity increases when VPA is used in children under 6 years old (Star et al., 2014). ALT is an important indicator for assessing liver function, in which elevated levels may indicate liver damage (Ghozzi et al., 2011). In this study, consistently, ALT was negatively correlated with VPA trough concentration (*r* = −0.103, *p* < 0.05), indicating that ALT is positively correlated with the concentration of VPA metabolites (Chen et al., 2012). In patients with strong metabolic capacity, especially children, VPA trough concentration is low, while the concentration of hepatotoxic metabolites is high, which may increase the risk of liver damage with elevated ALT (Zhao et al., 2020). However, in the pediatric patient population of this study, no cases of severe liver function damage were observed and ALT levels of most children remained within the normal range. This phenomenon can explain why the weight of ALT in monitoring VPA trough concentration was relatively low (Figure 2). Monitoring ALT levels is important for assessing and preventing liver damage.

In the GBRT, RFR, and SVR individual covariate models, the importance of the ten covariates varied to different extents (Figure 2). Daily dose, PLT, and RBC showed relatively consistent importance across these three models. However, the importance of VPA dosage form and UREA fluctuated significantly in these models. To balance the impact of each covariate on the output of the monitoring model and to enhance the role of important covariates, we decided to integrate these three independent models into an ensemble model to further improve the monitoring performance of the model. Our study results also confirmed this: the ensemble model showed consistent performance in both the training and testing datasets, indicating a proper model fitting. Additionally, compared with the three individual covariate models, this ensemble model demonstrated a clear superiority in monitoring performance (Figures 5, 6). The predictive results of this ensemble model are more in line with the clinical reality, which has profound practical value for clinical medication guidance and timely treatment.

The ensemble model showed significant differences in monitoring performance across different age groups. The in-depth analysis of the monitoring results of different age groups revealed that the model reported a relatively poor monitoring performance in patients under 3 years old but the best in the 3–10-years age group (Figure 7). The SHAP dependency plot illustrates the contribution of age feature values to the predictive outcome (Figure 3; Supplementary Figure S4). The data group for ages below 3 corresponds to SHAP values within the ±2 range (Supplementary Figure S4), indicating a lower contribution. Moreover, this data group has a significantly smaller sample size compared to other age subgroups, which may be one of the reasons for the relatively low relative and absolute accuracy of our model’s predictions. The metabolism of VPA is complex, involving three main pathways: mitochondrial-mediated β-oxidation, cytochrome P-450 (CYP450) catalyzed oxidation, and uridine diphosphate glucuronosyltransferase (UGTs) glucuronidation (Silva et al., 2008). Glucuronidation accounts for approximately 50% of VPA metabolism and is the most important factor affecting VPA blood concentration. However, UGTs gene polymorphism is developmentally regulated, with age affecting its metabolic rate, and young children have a higher metabolic capacity (Zhao et al., 2020). Some researchers believe this may be due to children having higher enzyme activity and a larger liver-to-body size ratio (Kearns et al., 2003). Therefore, the expression of UGTs gene polymorphisms in metabolic enzymes may significantly affect VPA metabolism, thereby affecting blood concentration and leading to significant inter-individual differences in blood concentration (Ghodke-Puranik et al., 2013). As this study did not include UGTs gene polymorphism as a covariate in the monitoring model, further research is needed to validate whether the poor monitoring effect observed in patients under 3 years old is related to the age-related differences in UGTs glucuronidation.

The ten covariates included in this study are not sufficient to fully represent all potential factors that may affect the monitoring of VPA trough concentration. Other factors that distribute VPA in the body, such as metabolic gene polymorphism and hypoalbuminemia, should be considered when constructing a monitoring model. To develop a mature monitoring model, more in-depth research work is needed. The current model has limited data outside the therapeutic window, which may, to some extent, limit the accuracy of model predictions. Especially in the patient population under 3 years old, the monitoring effect was not ideal, coupled with the relatively low rate of VPA trough concentration reaching the target range in this group, suggesting a need of due attention to this phenomenon. Therefore, follow-up studies need to acquire more data from the patient population under 3 years old to optimize the monitoring performance and generality of the model.

In translating the application of a ML-based ensemble model into clinical practice, several essential steps are involved (Zhu et al., 2022b): 1. collection of clinical data: gather more clinical sample datasets, incorporating a wider range of clinical feature variables including disease status, concomitant medications, and genetic polymorphisms, to further enhance the model’s clinical applicability. 2. external validation: require additional external validation datasets to assess the model’s accuracy and reliability in clinical settings. 3. drug clinical feature library: Establish a drug clinical feature library, similar to a disease database, capable of automatically capturing clinically relevant data for variables associated with valproic acid trough concentrations, especially data that can be aligned with testing times. 4. model transformation: transform the model into software, a website, or integrate it into electronic health records (EHRs) to enable automatic updates and dosage predictions, facilitating its use by clinical physicians. Correspondingly, potential barriers in clinical practice include (Tang et al., 2024): 1. quality and integrity of clinical data. 2. complexity and uncertainty of clinical data. 3. technical integration and compatibility. 4. ethical and privacy issues of clinical data.

## 5 Conclusion

The ensemble model proposed in this study, based on 3 ML techniques, can monitor the VPA trough concentrations in PEPs with a high degree of satisfaction. The advantage of this ensemble model lies in its ability to consider the importance of each covariate across different models, maximizing the utility of each covariate to enhance the overall monitoring performance. The monitoring results it yields are more clinically relevant, offering significant practical value for the individualized adjustment of clinical drug dosages and timely interventions in the precision treatment of PEPs.

## Data Availability

The original contributions presented in the study are included in the article/Supplementary Material, further inquiries can be directed to the corresponding author.
