# Supplementary material for: Monitoring of the trough concentration of valproic acid in pediatric epilepsy patients: a machine learning-based ensemble model
Source: Front Pharmacol. 2024 Dec 18;15:1521932. doi: 10.3389/fphar.2024.1521932 (PMC11688318; doi:10.3389/fphar.2024.1521932)
Supplement: Supplementary file 1 [file DataSheet1.docx]

**Supplementary Data**

**Table S1** The linear correlation between the VAP trough concentrations and the relevant covariates

| **Variables** | **Correlation Coefficient ^a^** |
| --- | --- |
| VPA Trough Concentration | 1.00 |
| Gender | 0.055 |
| Age | 0.159^**^ |
| Weight | 0.177^**^ |
| ALT | -0.103^*^ |
| AST | -0.039 |
| ALP | 0.026 |
| UREA | 0.172^**^ |
| CREA | 0.163^**^ |
| UA | -0.008 |
| Cys-C | 0.013 |
| ALB | -0.026 |
| GLO | 0.045 |
| WBC | -0.040 |
| RBC | -0.155^**^ |
| PLT | -0.381^**^ |
| NEUT | -0.034 |
| Dosage form | 0.244^**^ |
| VPA Daily dose | 0.344^**^ |
| Combination of drugs | 0.006 |

^a^ Spearman correlation coefficient
^*^*p* < 0.05, ^**^*p* < 0.01

Combination of drugs：levetiracetam, oxcarbazepine, lamotrigine, perampanel, clonazepam, or topiramate.

**Table S2** The multicollinearity test analysis of the variables.

| **Variables** | **Tolerance (TOL)** | **Variance inflation factor (VIF)** |
| --- | --- | --- |
| Age | 0.040 | 24.97 |
| Weight | 0.044 | 22.48 |
| ALT | 0.919 | 1.09 |
| ALB | 0.837 | 1.20 |
| UREA | 0.921 | 1.09 |
| CREA | 0.412 | 2.43 |
| RBC | 0.818 | 1.22 |
| PLT | 0.876 | 1.14 |
| Dosage form | 0.337 | 2.96 |
| VPA Daily dose | 0.467 | 2.14 |

**Table S3** Comparative performance of the three ML-based covariate models in the testing group (ages 3 to 10).

| **Group** | **Model** | **MAE** | **RMSE** | ***R^2^*** | **Relative accuracy^a^** | **Absolute accuracy^b^** |
| --- | --- | --- | --- | --- | --- | --- |
|  | GBRT | 9.70 | 12.64 | 0.63 | 84.7% | 77.2% |
| Training | RFR | 9.72 | 12.44 | 0.65 | 86.8% | 81.0% |
|  | SVR | 10.00 | 15.11 | 0.48 | 83.1% | 76.2% |
|  | GBRT | 11.64 | 14.01 | 0.49 | 83.3% | 68.8% |
| Testing | RFR | 11.71 | 14.37 | 0.47 | 87.5% | 68.8% |
|  | SVR | 11.74 | 15.06 | 0.41 | 85.4% | 68.8% |

**Table S4** The model performance metrics of the ensemble model (ages 3 to 10).

| **Group** | **MAE** | **RMSE** | ***R^2^*** | **Relative accuracy** | **Absolute accuracy** |
| --- | --- | --- | --- | --- | --- |
| Training | 9.58 | 12.68 | 0.63 | 83.6% | 80.4% |
| Testing | 11.23 | 13.94 | 0.50 | 85.4% | 66.7% |

**Table S5** Comparative performance of the three ML-based covariate models in the validation groups.

| **Group** | **Model** | **MAE** | **RMSE** | ***R^2^*** | **Relative accuracy** | **Absolute accuracy** |
| --- | --- | --- | --- | --- | --- | --- |
|  | GBRT | 10.45 | 13.86 | 0.36 | 75.0% | 71.9% |
| Validation 1 | RFR | 9.64 | 13.22 | 0.42 | 84.4% | 78.1% |
|  | SVR | 11.27 | 14.41 | 0.30 | 78.1% | 65.6% |
|  | GBRT | 11.26 | 14.10 | 0.30 | 75.0% | 71.4% |
| Validation 2 | RFR | 9.40 | 12.45 | 0.45 | 82.1% | 75.0% |
|  | SVR | 9.46 | 11.56 | 0.53 | 85.7% | 85.7% |

Validation 1, 32 in-hospital data samples.

Validation 2, 28 out-of-hospital data samples.

**Table S6** Comparison of the three approaches.

|  | **NONMEM** | **Bayesian** | **Machine Learning** |
| --- | --- | --- | --- |
| **Data Processing** |  |  |  |
| Flexibility | Can handle complex pharmacokinetic and pharmacodynamic models, but requires expertise to build models | Flexible, can integrate prior knowledge, but has certain requirements for data quantity and quality | Extremely flexible, capable of handling large-scale and complex datasets |
| Model Building | Requires model building based on pharmacokinetic theory | Can use prior knowledge and historical data to build models | Automatic learning and pattern recognition, no explicit theoretical model required |
| Parameter Estimation | Population-based parameter estimation | Parameter estimation considers prior distribution | Parameter estimation usually through optimization algorithms, such as gradient descent |
| **Expertise Required** |  |  |  |
| Demand | High, requires pharmacokinetic and statistical expertise | Moderate, requires understanding of Bayesian theory and statistics | Low to moderate, depending on the complexity of the algorithms and models used |
| Application Area | Mainly used in pharmacokinetic and pharmacodynamic research | Widely applied in statistical inference and machine learning fields | Widely applied in various fields, including image recognition, natural language processing, etc. |
| **Predictive Performance** |  |  |  |
| Accuracy | High, but dependent on the accuracy of model construction | Accuracy depends on prior knowledge and data quality | High accuracy, especially in large datasets |
| Prediction Range | Usually limited to pharmacokinetic and pharmacodynamic parameters | Can be extended to other fields based on prior knowledge | Can predict a wide range of outputs, not limited to specific fields |
| Model Validation | Requires visual and statistical validation | Need to check posterior distribution and model convergence | Requires cross-validation and hyperparameter tuning |


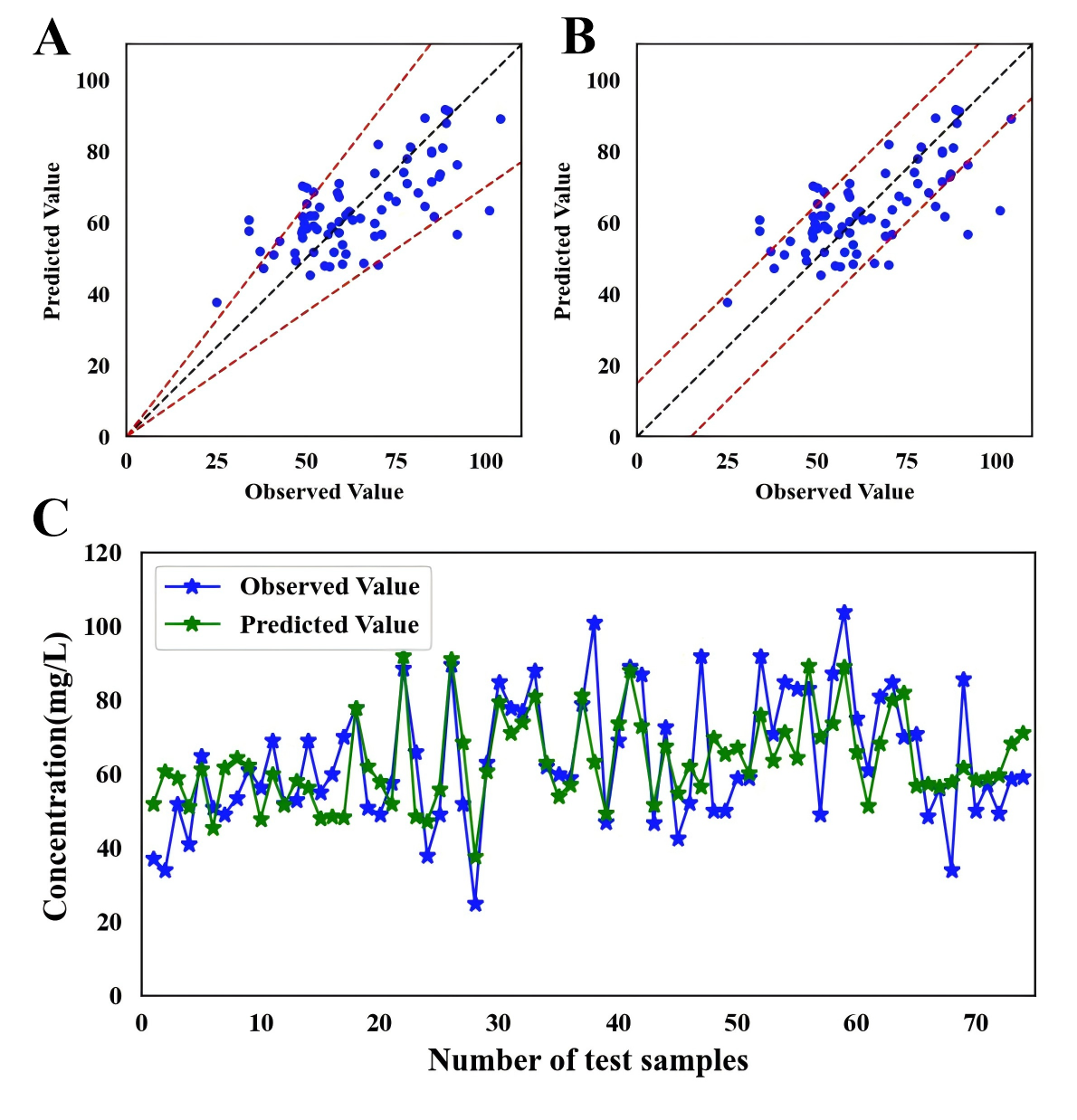


**Figure S1** The accuracy and prediction plot of the GBRT regression model. The blue dots represent the testing sample, with observed values on the x-axis and predicted values on the y-axis. (A) The blue dots between the dotted lines indicate that the predicted values were within ± 30% of the observed values (relative accuracy) and (B) the blue dots between the dotted lines indicate that the predicted values were within ± 15 mg/L of the observed values (absolute accuracy). (C) The blue dots indicate the observed values and green dots indicate the predicted values. The samples on the x-axis are ordered according to age, increasing from left to right in ascending order.


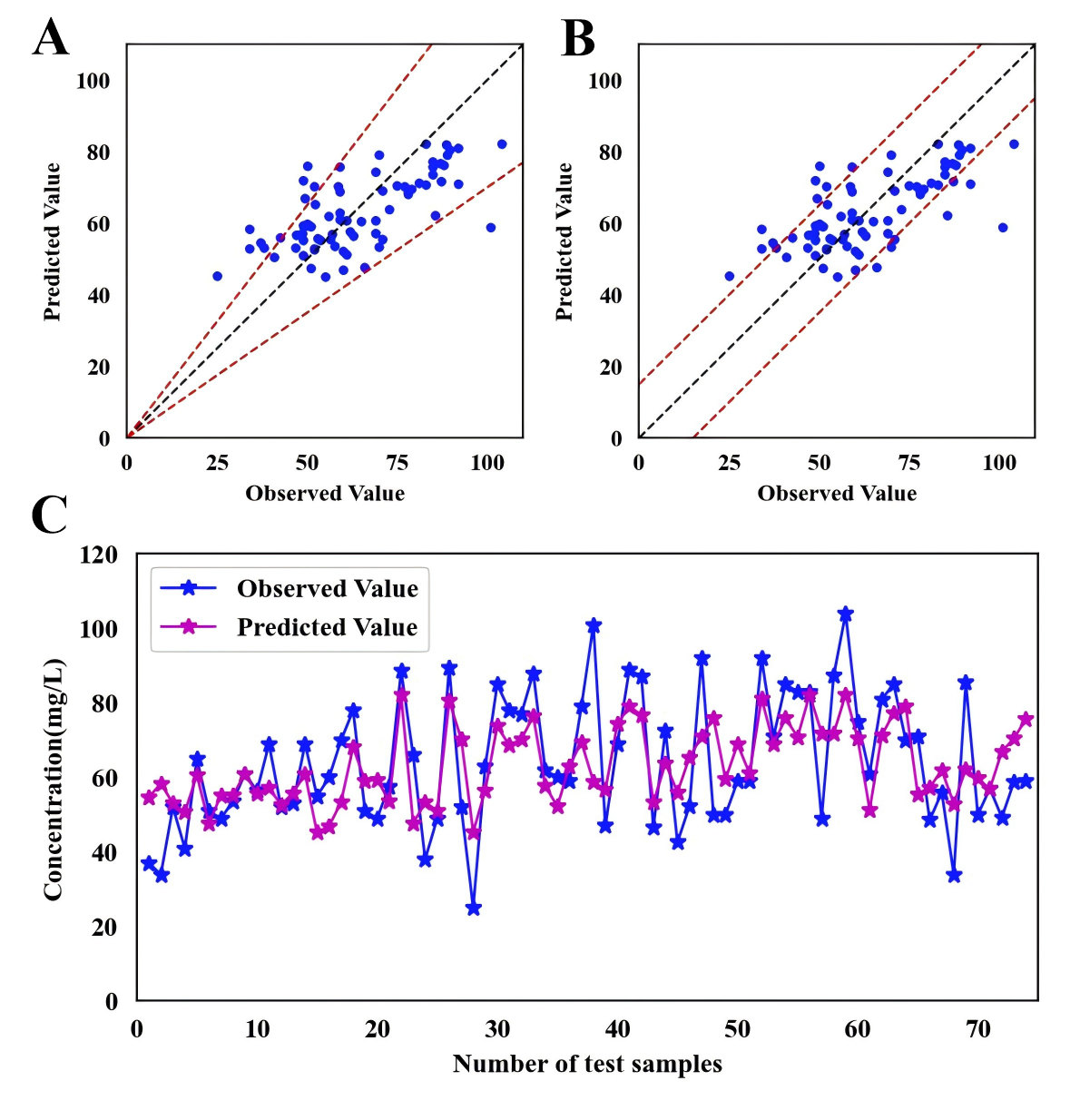


**Figure S2** The accuracy and prediction plot of the RFR regression model. The blue dots represent the testing sample, with observed values on the x-axis and predicted values on the y-axis. (A) The blue dots between the dotted lines indicate that the predicted values were within ± 30% of the observed values (relative accuracy) and (B) the blue dots between the dotted lines indicate that the predicted values were within ± 15 mg/L of the observed values (absolute accuracy). (C) The blue dots indicate the observed values and purple dots indicate the predicted values. The samples on the x-axis are ordered according to age, increasing from left to right in ascending order.


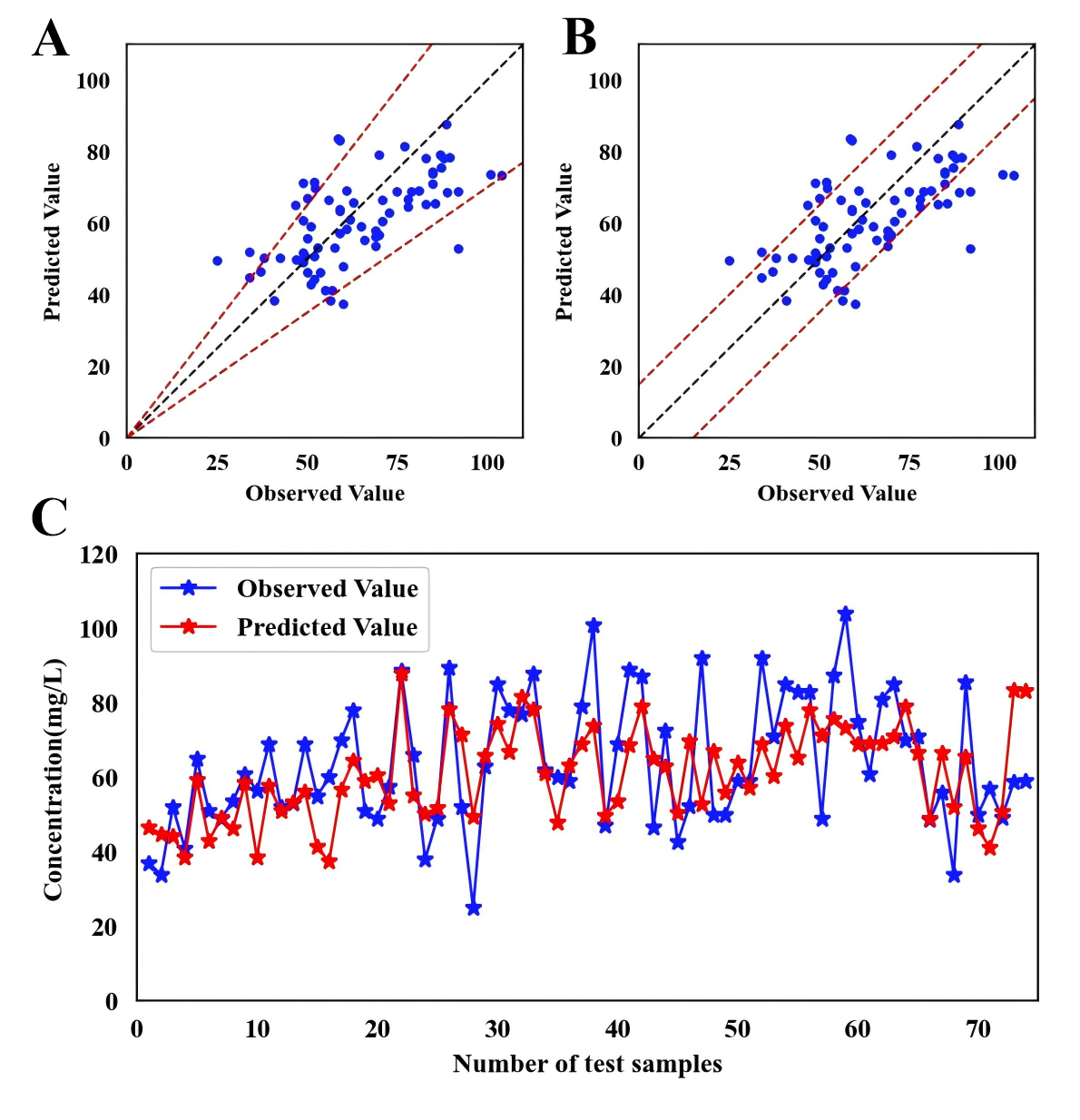


**Figure S3** The accuracy and prediction plot of the SVR regression model. The blue dots represent the testing sample, with observed values on the x-axis and predicted values on the y-axis. (A) The blue dots between the dotted lines indicate that the predicted values were within ± 30% of the observed values (relative accuracy) and (B) the blue dots between the dotted lines indicate that the predicted values were within ± 15 mg/L of the observed values (absolute accuracy). (C) The blue dots indicate the observed values and red dots indicate the predicted values. The samples on the x-axis are ordered according to age, increasing from left to right in ascending order.


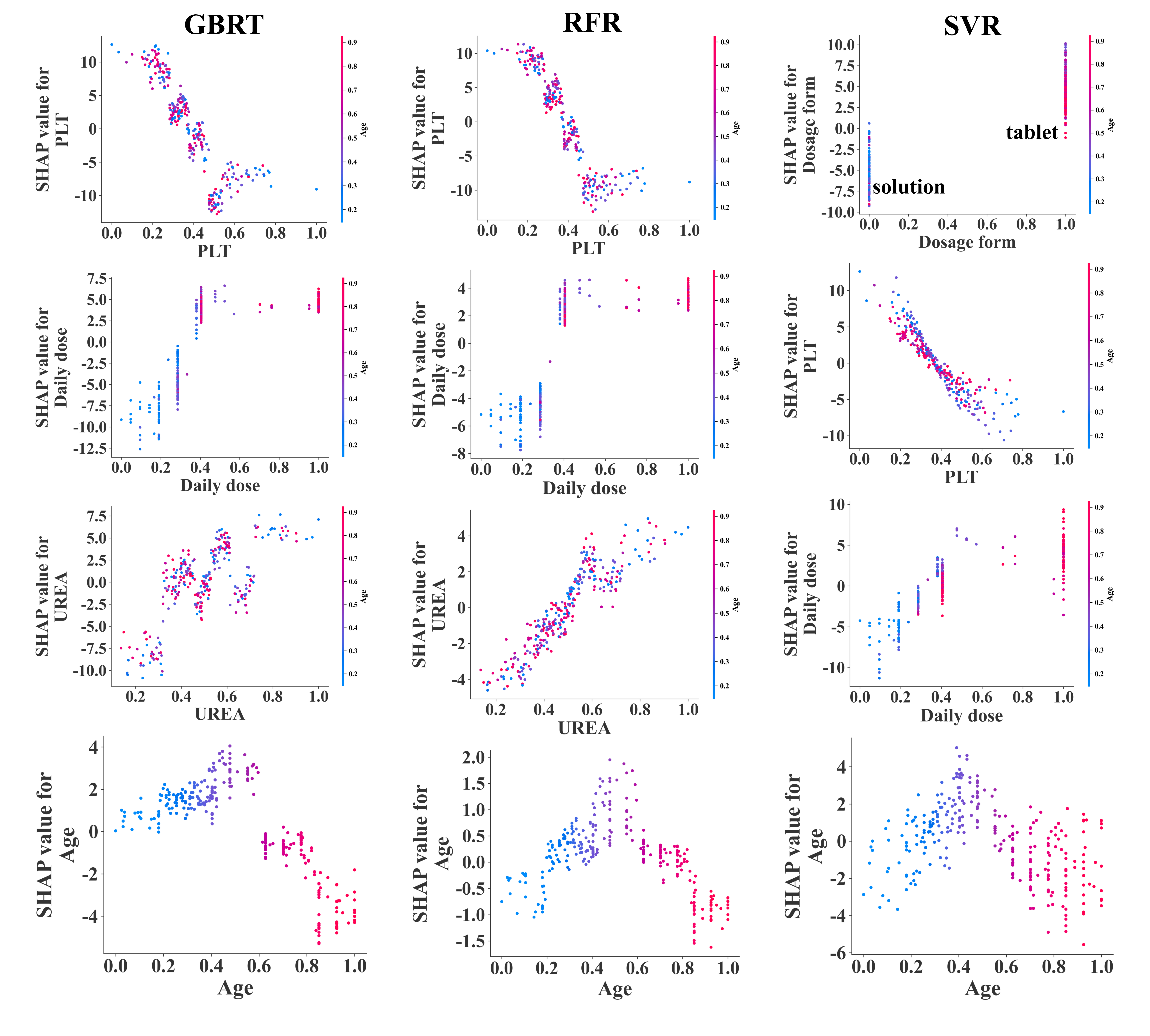


**Figure S4** SHAP dependency plots highlighting the significance of the top three variables across the three machine learning models: GBRT, RFR, and SVR. The color coding reflects the influence of the age, with a gradient from red indicating higher values to blue indicating lower values. These SHAP dependency plots illustrate the effect of relevant variables on the monitoring model's output. A SHAP value above 0 for a specific covariate suggests an increase in VPA trough concentration. For the VPA dosage form, 0 represents solution and 1 represents sustained-release tablet.
